# The Integration of Color-Selective Mechanisms in Symmetry Detection

**DOI:** 10.1038/srep42972

**Published:** 2017-02-23

**Authors:** Chia-Ching Wu, Chien-Chung Chen

**Affiliations:** 1 Department of Psychology, Fo Guang University, Yilan, Taiwan; 2 Department of Psychology, National Taiwan University, Taipei, Taiwan

## Abstract

We studied how the visual system detects multicolor symmetric patterns by manipulating the number of colors in an image in both isoluminance and luminance conditions. With a two-interval forced choice noise masking paradigm, we presented a noise mask in both intervals of each trial. A vertically symmetric target was randomly presented in one interval while a noise control was presented in the other. The task of the observers was to determine which interval contained the target. The target detection threshold was measured at various noise mask densities, which was found to decrease 1.4- to 2.5-fold as the number of colors in the image went up at median to high noise densities across different conditions. In addition, this color facilitation effect was greater in luminance conditions than in isoluminance conditions. Our data cannot be explained by the probability summation theory or simple signal-to-noise ratio. We therefore propose a computational model that incorporates a linear chromatic symmetry register, a nonlinear transducer response, noise manipulation and a multiple channel decision making process. This model suggests that the increment of the number of colors reduces the interference to the symmetry channels produced by noise, and in turn facilitates symmetry detection.

Symmetry is one of the most salient image features to be picked up by the visual system. A human observer can detect symmetry with great efficiency^1^ even with very brief exposure^2^,^3^,^4^. However, while a human observer can detect symmetry easily^1^,^2^,^3^,^4^,^5^, this detection requires complicated information processing and computation in the visual system^6^,^7^. By definition, a visual stimulus is symmetric if one part of the stimulus is a reflection of another part about an axis, called the symmetry axis. To determine whether an image is symmetric, the visual system first has to find correspondence between local features with either an idiosyncratic filter^8^,^9^,^10^,^11^,^12^,^13^,^14^,^15^ or reverse mapping^1^,^6^,^16^,^17^,^18^,^19^,^20^,^21^. Then, a higher-order mechanism takes the output of these early mechanisms and determines the orientation and location of the symmetry axis^6^,^16^,^17^.

Current models of symmetry perception work well with patterns in which symmetry is determined by a correspondence in one image property, such as luminance. However, many symmetric objects in everyday life, such as butterflies or birds, contain multiple cues signaling symmetry, and it is not clear how the visual system integrates those cues in determining the symmetry of these objects.

For the convenience of discussion, let us consider a symmetric pattern containing image elements of different colors, as shown in Fig. 1A,D,G, where the images are composed of dots either all in the same color (1-color symmetric pattern, Fig. 1A), in two colors (2-color symmetric pattern, Fig. 1D), or in four colors (4-color symmetric pattern, Fig. 1G). All of the above images contain the same number of dots. In each image, there are the same number of dots in each color. An observer should have no problem perceiving multicolor symmetry (e.g., Fig. 1D,G).

Wu and Chen^22^ measured the symmetry detection threshold of a symmetric target or signal (e.g., Fig. 1A) embedded in a noise mask (e.g., Fig. 1B) in a compound image such as Fig. 1C. They showed that a noise mask whose color was sufficiently similar to the target elevated the target threshold, while those of a different color did not. This suggests that, different chromatic information is processed by different color-selective symmetry channels in symmetry detection. Hence, the issue here is how the visual system integrates information across different channels for these colors.

In this study, we explored the role of multiple channels in symmetry detection. Symmetry detection has been shown to be based on the signal-to-noise ratio in the stimulus, rather than signal strength alone^1^,^18^,^19^,^20^,^21^. Hence, in our experiment we used a noise masking paradigm, in which a target, or signal, superimposed on a noise. A temporal 2IFC paradigm was used to measure the target density thresholds of symmetry detection. In each trial, a symmetric target was randomly presented in one of the two intervals while a balancing control was presented in the other. Both were embedded in a noise mask. The task of an observer was to indicate which interval contained the target. We manipulated the number of colors in the stimulus to measure the symmetry density threshold, or the density of signal required for an observer to achieve 75% accuracy in symmetry detection, at various noise densities. This allowed us to get the target threshold vs. noise density (TvD) functions for images with different numbers of colors, and observe the effect of the number of colors in the images on TvD functions, which in turn allowed us to investigate the properties of the mechanisms underlying symmetry detection^1^,^6^.

## Results

Figure 2A–C show the TvD functions for the isoluminance conditions. Figure 2A shows the isoluminant 1-color conditions; 2B, the 2-color conditions; and 2C, the 4-color conditions. Each row represents the TvD functions for one observer. The red symbols represent the data points of condition red (R); the blue symbols, condition blue (B); the green symbols, condition red-green (RG); the purple symbols, condition red-blue (RB); and the pink symbols, condition red-green-blue-yellow (RGBY). The smooth curves are fits of the model. The curves of condition R in the middle and right columns are the same as those in the left column. They were plotted in each column for comparison. Similarly, the curves of condition RG in the right column are the same as those in the middle column, also re-plotted for comparison.

For all conditions, the target density threshold increased with noise density. At medium to high noise densities (above −2.3 log units), the slope of the TvD functions showed no significant difference (*F*(4, 2) = 0.92, *p* = .50) across all five isoluminance conditions. The slope, averaged across all these conditions, was 1.21 in log-log coordinates and was significantly different from 1 (*t*(4) = 6.71, *p* = .003). For all three observers, there was no difference in threshold between the two 1-color conditions (R and B) (all *t*(8) < 1.44, *p* > .19). We thus pooled these two conditions together for the subsequent data analysis.

To better assess the effect of the number of colors, we calculated the threshold difference between the three multicolor conditions (RG, RB, and RGBY) and the pooled 1-color condition (R and B), shown in Fig. 3A. The green symbols in Fig. 3A denote the threshold difference between condition RG and the pooled 1-color condition, averaged across the three observers; the purple symbols, between condition RB and the pooled 1-color condition; and the pink symbols, between condition RGBY and the pooled 1-color condition. The smooth curves are the predictions of the model discussed below. As the figure shows, the threshold reduction in the multicolor conditions increased with the increment of the noise density at low noise densities (equal to or less than −2.3 log units) up to 0.27 log units or a 1.86-fold change. At median to high noise densities (above −2.3 log units), the threshold reduction was relatively constant. Except for two points, the magnitude of the threshold reduction was between 0.2 to 0.3 log units (or a 1.6 to 2-fold increase) at median to high noise densities for all three multicolor conditions.

Figure 2D shows the TvD functions for the four luminance conditions. Each row represents the TvD functions for one observer. The red symbols represent the data points of condition white (W); the green symbols, condition white-black (WK); the purple symbols, condition white-red (WR); and the pink symbols, condition white-black-red-green (WKRG). The smooth curves are fits of the model discussed below. Similar to the isoluminance conditions, the target density threshold increased with noise density. Again, at noise densities above −2.3, the slope of the functions for all four conditions showed no significant difference (*F*(3, 2) = 0.11, *p* = .95). Averaged across all conditions, the slope of the TvD functions within this range reached an average of about 1.20 in log-log coordinates, which was significantly different from 1 (*t*(3) = 11.44, *p* = .0014).

We also calculated the threshold difference between the three multicolor conditions (WK, WR, and WKRG) and the 1-color condition (W) to assess the effect of the number of colors. The green symbols in Fig. 3B represent the threshold difference between conditions WK and W, averaged across three observers; the purple symbols, between conditions WR and W; and the pink symbols, between conditions WKRG and W. The smooth curves are the predictions of the model discussed below. As the figure shows, the threshold reduction in the multicolor conditions increased with the increment of the noise density at low noise densities (equal to or less than −2.3 log units) up to 0.24 log units or a 1.7-fold change. At median to high noise densities (above −2.3 log units), the threshold reduction was relatively constant. The magnitude of threshold reduction across the three multicolor conditions was from 0.14 to 0.4 log units (or a 1.4- to 2.5-fold change) at median to high noise densities. In this range, the threshold reduction in the 4-color condition (*M* = 0.33, *SD* = .06) was larger than in condition WK (*M* = 0.19, *SD* = .06, *t*(3) = 7.82, *p* = .002) and condition WR (*M* = 0.17, *SD* = .05, *t*(3) = 4.00, *p* = .01). This 4-color facilitation effect was even larger than in the isoluminance condition (0.21 to 0.31 log units, *M* = 0.24, *SD* = 0.05; *t*(3) = 4.73, *p* = 0.009).

For a more quantitative analysis of the result, we fit a multiple channel symmetry detection model derived from Chen & Tyler^6^ to the data (Fig. 4). The first stage of this model contains a band of color-selective symmetry registers which are excited by a symmetry pair of a specific color. The excitation from all symmetry pairs about a given symmetry axis is linearly summed to produce the excitation of that register, *E*. The linear register is followed by a nonlinear response operator. The response of each symmetry channel is the excitation of the corresponding register raised by a power, *p*, and divided by a constant, *z*, plus a divisive inhibition input, *I*, which is a nonlinear sum of the excitations of all relevant linear registers to all the components, symmetric and non-symmetric, in an image. That is, the response of the *j*-th channel is


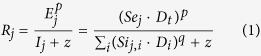


where *Se*_*j*_ is the excitatory sensitivity of the *j*-th channel to the symmetric component or target; *Si*_*j,i*_, the inhibitory sensitivity of the *j*-th channel to the image component *I; D*_*t*_, the density of the symmetric component; and *D*_*i*_, the density of the *i*-th image component. The threshold is achieved if the difference between the signal and the noise intervals in a decision variable, which is the ratio of Minkowski summation of responses divided by the amount of noise, reaches a critical value. The detail derivation and implement of the model is described in the Method section.

The model fits are shown as smooth curves in Fig. 2. The model explains 98–99% of all variability in thresholds across three observers. The root mean square error (RMSE) is between 0.08 to 0.09 log units across observers, on par with the average standard error of measurement.

As shown in Table 1, the inhibitory sensitivity to the noise component of the preferred color (*Si*_*m*_) varied with the type of color modulation (i.e., isoluminance and luminance) and the number of colors (i.e., 1-, 2-, and 4-color) in an image. Since the inhibition from the noise component to a channel is the product of the inhibitory sensitivity and the noise density (*D*_*m*_), we can estimate the amount of inhibition from a noise component of a specific color in an *n*-color condition as *Si*_*m*_ × *D*_*m*_/*n*. As illustrated in Fig. 5A, in both luminance and isoluminance conditions, the inhibition from noise decreased with the increment of the number of colors in an image (*F*(12,198) = 2.19, *p* = .001). That is, noise had less effect in a multicolor display than a single-color display. In addition, the inhibition from the luminance noise was less than from the isoluminance noise. This effect is small but significant: if we constrain the inhibition parameters to be the same for luminance and isoluminance conditions, the sum of square of error (SSE) of the model increases significantly even if we take the number of parameters into account (*F*(9,198) = 4.23, *p* < 0.0001). Notice that, for visualization purposes, we divided the inhibition in each condition by that of the 1-color isoluminance condition for each observer.

The additive constant *z* also decreased with the number of colors in an image, shown in Fig. 5B, which illustrates the relationship of the normalized *z* value, averaged across three observers, with the number of colors.

## Discussion

In this study, we investigated the effect of the number of colors on symmetry detection. We measured the target density thresholds for symmetry detection in 1-, 2-, and 4-color patterns in the presence of noise masks of various densities. Our results showed that an increment in the number of colors in the stimuli facilitated symmetry detection. The target density threshold decreased with the increment of the number of colors, especially at median to high noise densities. This facilitation effect was larger in the luminance conditions than the isoluminance conditions.

The decrease of the detection threshold with the number of colors in a display at a given noise level can be explained by the reduction of inhibition from noise when there are more colors (Fig. 5A). This reduction in noise interference can be explained in terms of the correspondence problem. As in the original correspondence problem in stereopsis^23^, to perceive symmetry is to find correspondence between two parts of an image. Any dot in the display can match to any other dot of the same color. However, only one of these possible matches is consistent with the perceived symmetry pattern while others are false matches. The difficulty here is that there is no *a priori* reason to believe any particular match is true.

As illustrated in Fig. 5C, in a 1-color image, a dot can pair with any of the *n*-1 dots in the image and thus there are *n*(*n*-1) = *n*^2^ - *n* possible dot pairs in total. For each dot pair, one can always find an axis bisecting the connection line between the two dots. Hence, each of those matches would produce an excitation in certain symmetry channels and in turn contribute to the divisive inhibition term. However, since each dot can only pair with another dot of the same color^22^, in a 4-color display, for example, there are only *n*/4-1 candidate pairs for each dot and, in turn, a total of (*n*/4) × (*n*/4 - 1) × 4 = *n*^2^/4 - *n* pairs. Fewer dot pairs mean fewer channels being excited and contributing to the divisive inhibition term. Hence, the divisive inhibition from a 4-color pattern would be less than that of a 1-color pattern.

Some may wonder why the observers should still consider the possible dots in all different orientations given that our observers had the knowledge that the symmetry axis was always vertical. The reason is that the early symmetry registers just passively operate on the image and are excited by whatever dot pairs that fit their sensitivity profile. Thus, at this stage, the visual system still takes all possible pairs into account. Only at the decision stage, the prior knowledge allows the observer to monitor just the target channel and ignore others. This notion is supported by Chen & Tyler^6^, who showed that the symmetry detection threshold still increased with noise level even when the observer was cued the symmetry axis orientation, implying a noise effect that is not affected by prior knowledge about the symmetry axis.

We also found that the threshold reduction for the multicolor conditions was larger in luminance than isoluminance conditions. This implies that the response of the luminance channel, or a set of luminance channels, differs from that of the isoluminance channels. The model fitting results also suggest that the luminance channels are more noise resistant; the inhibition from the luminance noise was less than from the isoluminance noise and, in turn, there was a greater response from the luminance channels. This lower inhibition corresponds to better symmetry detection performance in the luminance conditions. This may result from a smaller inhibitory sensitivity to the contribution from other channels.

The additive constant *z* in our model also decreased with the number of colors in an image (Fig. 5B). The additive constant is known to change with adaptation^24^. It is possible that, compared with the multicolor conditions, the stronger signal in each symmetry channel in the 1-color conditions leads to a greater adaptation effect in the visual system.

In sum, our data can be accounted for by these parameter changes. At low noise levels, the inhibition term from the external noise is negligible when compared with the additive constant *z*. The denominator of the response function (equation (4) in the Method) is mainly dominated by the constant *z*. Hence, the decrease of this constant accounts for the threshold reduction, rather than the threshold increment predicted by probability summation theory at low noise levels in the 2- and 4-color conditions. As the external noise level increases, the denominator of the response function is gradually dominated by inhibition from the external noise. Hence, the reduction of this noise-dependent inhibitory term produces a threshold reduction at median to high noise levels. The reduction of this inhibitory term with the increment of the number of colors therefore reduces the threshold more at median to high noise levels when an image contains more colors, as observed in Fig. 3. The greater inhibitory term from the isoluminance noise than from the luminance noise also accounts for better performance in the 4-color luminance conditions than in the isoluminance conditions at median to high noise densities.

Note that we acknowledge that, as the number of colors increases, there are also fewer signal dots in each color (*E*_*j*_ in equation (2) in the Method) and, in turn, less excitation in each channel since the excitation is proportional to the density of the signal dots. This reduction in the numerator of the response function is obviously more than compensated for by the reduction in the denominator since the threshold reduction is consistent with the change of inhibition term.

**Figure 1:**
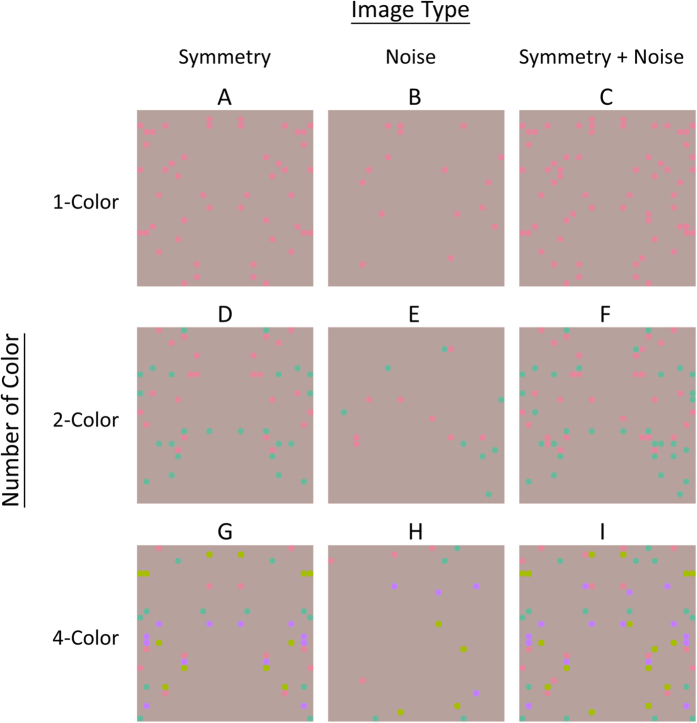
Example of the stimuli. (**A**) is a red symmetric target; (**B**), a red noise mask; and (**C**), a compound image in which a red symmetric target (**A**) is embedded in a red noise mask (**B**). (**D**) is a symmetric target composed of red and green dots; (**E**), a noise mask composed of red and green dots; (**F**), a compound image in which a red-green symmetric target (**D**) is embedded in a noise mask of the same colors (**E**). (**G**) is a symmetric target composed of red, green, blue and yellow; (**H**), a noise mask and (**I**) is a compound image with a symmetric target (**G**) embedded in a noise mask of the same colors (**H**).

**Figure 2:**
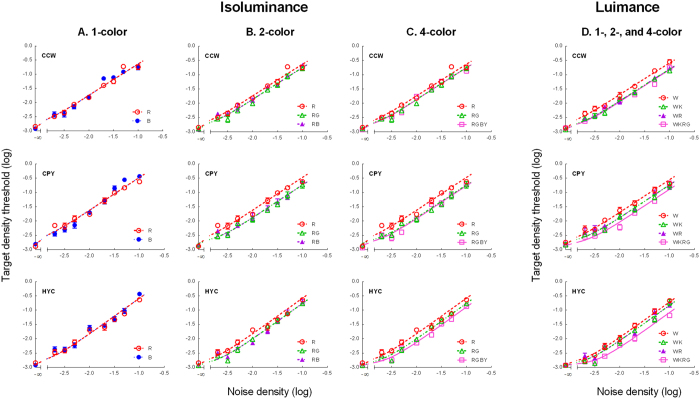
Target threshold vs. mask density (TvD) functions. (**A**–**C**) The TvD functions for the isoluminance conditions. Each panel represents the data from one observer. (**A**) 1-color conditions. The red and blue symbols represent the data points of the R and B conditions respectively. (**B**) 2-color conditions. The green and purple symbols represent the data points of the RG and RB conditions respectively. (**C**) 4-color condition. The pink symbols represent the data points of the RGBY condition. The smooth curves are fits of the model. The curves of condition R in the middle and right columns are the same as in the left one, and the curve of RG in the right column is the same as in the middle one, re-plotted for comparison. (**D**) The TvD functions for the luminance conditions. Each panel represents the data from one observer. The red, green, purple, and pink symbols represent the data points of the W, WK, WR, and WKRG conditions respectively. The smooth curves are fits of the model.

**Figure 3:**
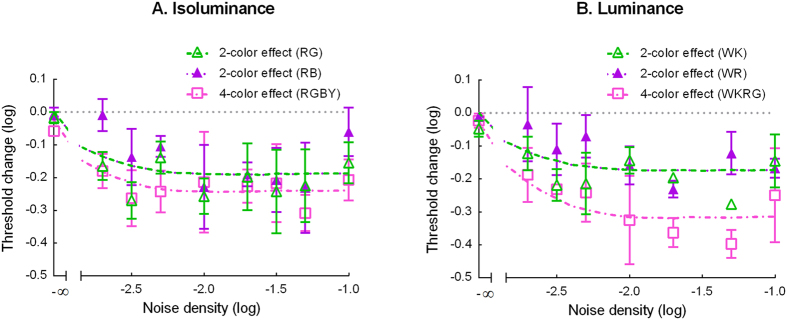
The average threshold change produced by 2-color and 4-color conditions. (**A**) Isoluminance condition. The average threshold change produced by the two 2-color (RG and RB, green and purple symbols) and the one 4-color (RGBY, pink symbols) isoluminance conditions at different noise densities. (**B**) Luminance condition. The average threshold change produced by the two 2-color (WK and WR, green and purple symbols) and the one 4-color (WKRG, pink symbols) luminance conditions at different noise densities. The error bar is the standard error. The smooth curves are the predictions of the model.

The key component of our model is the nonlinear response function. Without this nonlinear response function, the whole is reduced to the traditional probability summation model (equation (6) in the Method) based on the signal-to-noise ratio (equation (8) in the Method) computation. The core of the probability summation model is that a multiple channel system can detect a target if the response of any of its channels reaches a criterion^25^,^26^,^27^,^28^. Hence, a system with more channels would detect a target more easily than one with fewer channels because, due to random variation, it has a greater chance of having at least one channel with a response exceeding a criterion^27^,^29^. In our study, different colored symmetric components of a pattern elicited response in different channels. Hence, the visual system needs to monitor more channels in multicolor conditions than in 1-color conditions. Suppose that an observer can detect symmetry if there are *k* symmetry pairs with a certain probability in a 1-color symmetric image (such as Fig. 1A). In a 2-color pattern (Fig. 1D), an observer can detect symmetry in either of the two colors and has a greater chance of detecting symmetry if there are also *k* symmetry pairs in each color. Thus, to reach the same level of detectability, according to the fourth-power rule of probability summation^25^,^27^,^28^,^29^, each color component would contain 0.84*k* symmetry pairs since ((0.84*k*)^4^ + (0.84*k*)^4^)^0.25^ = *k*. Thus, the symmetry detection threshold for a 2-color symmetric pattern should be 1.68 times (i.e., 0.23 log unit increase) that of the 1-color pattern (0.84*k* × 2 = 1.68*k*) when there is no noise mask. Similarly, the threshold for a 4-color symmetric pattern (Fig. 1G) should be 2.83 times (i.e., 0.45 log unit increment) that of the 1-color pattern. When there is a noise mask, the threshold is achieved when the signal to noise ratio (i.e., the ratio of symmetry pairs to unpaired dots) reaches a certain level. Given the same rationale described above, the probability summation theory would predict that the threshold for the 2-color pattern at a given noise density should be about 0.84 times, or 0.076 log units, lower than that for the 1-color pattern while the threshold for the 4-color pattern should be about 0.15 log units lower than that for the 1-color pattern (See Supplementary Discussion for the details). Such predictions largely underestimate the threshold reduction, especially at medium to high noise densities, for both the 2-color (0.06 to 0.28 log units of threshold reduction across different conditions) and the 4-color (0.21 to 0.40 log units of threshold reduction) conditions. The prediction of the probability summation model and our data are presented in Fig. 6A, in which the left panel shows predictions for isoluminance conditions and the right for luminance.

The signal-to-noise ratio aspect of the model (equation (8) in the Method) is similar to the signal-to-noise ratio (SNR) or “weight-of-evidence” model for symmetry detection^1^,^18^,^19^,^20^,^21^. In such a model, the visual system might simply analyze the spatial relationships among dots and determine an image to be symmetric if a sufficient proportion of the symmetric pairs support it. Notice that, the original WOE model^18^,^20^,^21^ treats all dots the same and thus, in its original form, it would be difficult to apply this model to our multiple-color display experiment. However, the core concept of SNR is quite relevant to the current study. One might argue that our multiple color effect could be explained by the SNR in the channels since there are fewer spurious symmetric pairs in the noise in the multicolor conditions and thus, in a two-interval forced choice (2IFC) task, the difference between the target interval and the non-target interval is greater than in the 1-color conditions. This simple model, while it predicts a lower threshold for the multicolor conditions, severely underestimates this threshold reduction since the effect of spurious symmetric pairs is very small, especially at low noise densities. We fit this model to the data, shown in Fig. 6B. The dashed curves in Fig. 6B represent the prediction of the SNR model. The prediction obviously deviates from our data.

The facilitation effect in the multicolor conditions also runs counter to the prediction of the color-blind symmetry mechanisms^30^,^31^. If symmetry detection is mediated by such mechanisms, all colors would be treated the same by the visual system. Thus, there should be no difference between 1-, 2- and 4-color conditions. Our result is also inconsistent with some psychophysical theories of multiple channel integration. A linear summation model, in which the visual system simply sums symmetry information across different color-selective symmetry channels, obviously cannot explain our result. In this model, the visual system can detect symmetry if there are enough symmetry pairs in the image regardless of their colors. Hence, it would also predict no difference between the TvD functions for 1-, 2- and 4-color patterns, which contradicts our data.

Some might argue that the color-blind symmetry mechanisms with attention switch proposed by Pashler and his colleagues^30^,^31^, can explain our data. They presented their observers with patterns composed of colored squares arranged to be either completely symmetric about a vertical axis or with one or two pairs of corresponding squares mismatched in color. They showed that the response time for the observers to judge whether a pattern was symmetric was longer when the pattern contained four colors than when the pattern contained only two colors. Hence, they suggested that their result could be explained by color-blind symmetry mechanisms, guided by attention shift, which assess symmetry sequentially from one color to the other. Color symmetry is registered when every matching process is successful.

It is not clear how this model, derived from response time data, can explain the result of a threshold experiment like ours, since the decision criteria used in these two types of experiments are quite different. Here, we explore some possibilities. First of all, the color information of individual channels must be present at the decision stage. The symmetry detection mechanism cannot be color blind. Otherwise, there would be no difference in detection threshold for 1-, 2- or 4-color patterns. In our model, the decision stage monitors individual color selective channels and thus is influenced by the color difference in the stimuli. Pashler *et al*.‘s model also has the decision stage receiving inputs from different colors. However, they suggested that the information from the individual channels is lost and thus the symmetry mechanisms are color blind.

Furthermore, Pashler *et al*. suggested that the symmetry computation would not be complete until the matching process in each channel was finished. This suggests that symmetry detection is limited by the channel with the minimum symmetry response. This decision criterion is very different from what one would expect in a detection experiment, in which the stimulus is detectable if any channel can detect it and thus the performance is determined by the channel with the maximum response. The latter is actually a version of the probability summation model discussed above. As we have seen (see Fig. 6A), the probability summation model, without nonlinear response functions, underestimates the reduction of threshold when the number of colors increases. A model limited by the minimum response would predict higher thresholds for multicolor conditions and thus further underestimate the threshold reduction. Thus, it is unlikely Pashler *et al*.‘s model can explain our results.

Recently, Gheorghiu *et al*. found that there was no difference in symmetry detection performance between a “random-segregated” condition, in which the color of the symmetry signal was different from the noise, and a “non-segregated” one, in which the color of the symmetry signal was the same as the noise, similar to our study^32^. They argued that their result provides evidence against the probability summation theory, which would predict better detection performance in their “random-segregated” condition than the “non-segregated” one. Also, they found that symmetry detection performance was better in “non-segregated” and “anti-symmetry” conditions (note that their stimuli were not really anti-symmetry but position-matching; in anti-symmetry, the corresponding locations are of opposite luminance or opposite colors). Hence, they concluded that the symmetry mechanism is not color selective, but is sensitive to color correlation. However, their stimulus presentation was quite long (500 ms). Given that a human observer can detect symmetry in less than 100 ms^1^,^5^, this long duration may encourage interference from higher cognitive processes such as attention, visual search or strategy. Furthermore, their stimuli, unlike ours, contained dots near the symmetry axis. This encouraged their observers to use only local pairs near the axis for symmetry judgement.

Many studies showed that it is more difficult for a human observer to detect anti-symmetry than symmetry^30^,^32^,^33^,^34^,^35^,^36^,^37^. In our model, the symmetry detection mechanism contains registers that are activated by a symmetry pair, or a mirroring spatial variation at corresponding locations in the visual field across a symmetry axis. When the two corresponding locations do not have a matching spatial variation, they produce weaker or no activation. Hence, our model does predict that it would be more difficult to detect an anti-symmetric pattern. However, there are also studies showing that an observer can easily detect an anti-symmetric pattern when it has a low density^7^,^38^ or is composed of small elements^34^. These results suggest that, for a sparse display, the visual system may be able to utilize either broadly tuned mechanisms that can pick up signals with opposite luminance or color polarities or mechanisms that receive a full-wave rectified signal from the color opponent channels for symmetric detection. Our model does not contain those mechanisms. The mechanism underlying sparse anti-symmetry pattern detection is still unclear. Our experiment was not designed to study such patterns. Hence, at this point, our model can only offer a limited explanation on sparse anti-symmetry detection.

Wagemans *et al*.^16^,^17^ reported that the existence of a higher order structure (that is, not just dot pairs, but also the relationship between dot pairs conforms certain regularity) can facilitate symmetry detection. To account for their result, they proposed a model in which the global structure could help the formation of dot pairs for the desirable symmetry axis orientation and that the undesirable dots pairs would be eliminated from further processing. This model allowed them to explain many regularity phenomena, such as skewed symmetry. However, it is unclear what is the effect of noise dots in their model. It would be difficult for their model to account for our results, which showed that the detection threshold increased with noise, suggesting that noise dots did play a role in symmetry perception.

## Conclusion

In sum, our results show that the increment of the number of colors in an image facilitates symmetry detection. The visual system monitors color-selective symmetry channels whose color selectivity matches the colors of the symmetric pattern in order to detect symmetry. The more colors an image contains, the more symmetry channels need to be monitored. In this case, both the inhibition term and the additive constant decreased with the number of colors in an image. The inhibition reduction implies a reduction in the interference to the symmetry channel produced by noise, while the decrement of the additive constant implies a reduction of the adaptation effect in the visual system. These two factors account well for the enhancement of symmetry detection performance with the increment of the number of colors in an image.

## Method

### Ethics statement

This study was approved by the IRB of National Taiwan University Hospital (#200912065R, approval date: 28^th^ Jan 2010) and followed the guidelines of the Helsinki Declaration. Written informed consent was obtained from each participant.

### Subjects

Three observers participated in this experiment. Among them, CCW was one of the authors of this paper while the other two were naïve to the purpose of the experiment. All observers had corrected to normal (20/20) visual acuity.

### Apparatus

The stimuli were presented on a 24-inch LCD monitor controlled by a Macintosh computer via a Radeon 7200 graphics board which provided 10-bit digital-to-analog converter depth. The LCD monitor was calibrated with a PhotoResearch PR655 radiometer for both luminance and chromaticity. The display had a mean luminance of 76.81 cd/m^2^ and mean chromaticity at (0.33, 0.33) in CIE 1931-xy coordinates. The refresh rate of the monitor was 60 Hz. The viewing distance was set such that each pixel made up 1′ of visual angle.

### Stimuli

The stimuli were composed of dots randomly distributed in a 46 (W) by 40 (H) grid system. The width of each cell was 0.21° visual angle. The display had a 9.9° visual angle extent. The position of each dot was jittered within the cell. The purpose of using the grid system was to avoid overlapping dots within one image.

Each dot was defined by an 8th-power Gaussian function, or ***K***(*x, y*) = ***BG + BG***·***C***·exp(−(x^8^/2σ^8^ + y^8^/2σ^8^)) where x and y were the distances in degrees from the fixation point; σ  = 0.11° was the space constant; BG was a 3 by 1 vector which specified the cone excitation coordinates of the background; C was a 3 by 1 cone contrast vector^37^ which specified the color modulation.

The cone contrast vector ***C*** = [*C*_L_, *C*_M_, *C*_S_] was a vector with three elements. Among them, the L-cone contrast, *C*_L_, was defined as ΔL/L_0_ where L_0_ was the L-cone excitation produced by the background and ΔL = L − L_0_ where L was the L-cone excitation to the center of a dot. If there was a decrement in cone excitation, the cone contrast was negative. The M-cone and S-cone contrasts, denoted by *C*_M_ and *C*_S_ respectively, were defined similarly. Cone excitations were the product of the power spectral distribution of the light and the estimated spectral sensitivity functions of the corresponding cones^40^.

Each cone contrast vector can be separated into two parts: a scalar value for contrast and a normalized cone contrast vector, ***C***/||***C***||, where||***C***||denotes the length of the vector ***C***. In our experiment, the normalized cone contrast vectors were [0.577, 0.577, 0.577] and [−0.577, −0.577, −0.577] for white and black respectively, [0.416, −0.909, 0] for red, [−0.416, 0.909, 0] for green, [0, 0, 1] for blue, and [0, 0, −1] for yellow. Note that the normalized cone contrast vectors for isoluminant stimuli were all orthogonal to the CIE2007 luminous efficiency function V_λ_ 41, which corresponded to the normalized vector [0.853, 0.522, 0].

The contrast of a stimulus was defined as *c = *(*C*_L_^2^ + *C*_M_^2^ + *C*_S_^2^)^0.5^/3^0.5^. This measure was proportional to the square root of the cone contrast energy and varied between 0 and 1. Contrast was expressed in dB re 1 which equaled 20 log_10_ *c*. In the experiment, the contrast of each stimulus was set at three times the threshold, or 9.54 dB increments, of that stimulus for each observer, to control the salience of each color. The experiment for threshold measurement is described in Supplementary Method. Once the contrast of each color was determined, we asked each observer to judge the salience of each color in each multicolor display. The observers reported equal salience in all 18 colors (6 colors × 3 observers).

For better visualization, one can also represent the color of a stimulus as a point in a polar cone contrast space^42^,^43^,^44^ with the distance from the origin to the point representing contrast; elevation, luminance; and azimuth, hue. In this space, the elevations −90° and +90° represent black and white respectively. The azimuth angles 0°, 180°, 90° and 270°, with zero elevation, represent red, green, blue, and yellow respectively. Table 2 lists polar representations of our stimuli and their corresponding cone contrast vectors.

In each trial, the stimulus consisted of a vertically symmetric target or a balancing control, superimposed on a noise mask. The purpose of the balancing control was to balance the local statistics in the image, that is, to ensure that the two intervals in a trial always had the same number of dots and therefore, an observer could not use the quantity of dots for the task. Both the balancing control and the noise mask were composed of random dots. The density of the noise masks was from 0 to 10%, or −∞ to −1 log unit, in which the density was defined as all the noise elements divided by the number of the grids the elements were distributed over; 46 × 40 in this case. In the symmetric target, half of the image was a reflection of the other half about a vertical axis. That is, a pixel at position (x, y) of the symmetric target *I* had the property *I*(x, y) = *I*(−x, y). The target density was defined as the proportion of the grids that contained symmetric elements. The balancing control in the same trial had the same density. The densities of the target and the balancing control were the same but both varied from one trial to another according to the experimental procedure described below. To prevent observers from using local information near the axis to make a judgment, no dot was presented in the region 0.7° to the left and right of the symmetry axis.

There were five isoluminance and four luminance conditions in this experiment. In each condition, the target, balancing control and noise mask were the same colors. The five isoluminance conditions were red (R), blue (B), red-green (RG), red-blue (RB), and red-green-blue-yellow (RGBY). The conditions R and B were 1-color conditions. All the stimuli in the condition R were red, and those in the condition B were all blue. The conditions RG and RB were 2-color conditions. All the stimuli in the condition RG contained an equal density of red and green dots, and those in the condition RB contained red and blue dots. The condition RGBY was the 4-color condition. The stimuli contained red, green, blue, and yellow dots with equal density. The four luminance conditions were white (W), white-black (WK), white-red (WR), and white-black-red-green (WKRG). Similarly, all the stimuli were white in the condition W, white and black in the condition WK, white and red in the condition WR, and white, black, red, and green in the condition WKRG.

Figure 1 shows examples of our stimuli. The first row in Fig. 1 (Fig. 1A–C) shows examples of the 1-color condition R: Fig. 1A is a red symmetric target; Fig. 1B is a red noise mask; Fig. 1C represents a red symmetric target (Fig. 1A) embedded in a red noise mask (Fig. 1B). Similarly, the second row (Fig. 1D,F,G) shows a symmetric target, noise mask, and compound image in the 2-color condition; and the third row (Fig. 1H–J) the 4-color condition.

### Procedures

A temporal 2IFC paradigm was used to measure the target density thresholds of symmetry detection. In each trial, a symmetric target was randomly presented in one of the two intervals while a balancing control was presented in the other. Both were embedded in a noise mask.

The stimulus duration was 233 ms and the inter-stimulus interval (ISI) was 600 ms. An audio tone indicated the beginning of each interval. The task of the observers was to judge which interval contained a symmetric target. The observers were instructed to spread their attention to the whole pattern and to avoid focusing on a particular color or a specific region. They were also informed that the symmetry axis was vertical. An audio feedback was provided for the response. We used the PSI threshold-seeking algorithm^45^ to determine the density of the target and balancing control in each trial and measured the target density threshold at 75% correct level. There were 40 trials for each threshold measurement. Each datum point reported was an average of four to eight repeated measurements. The isoluminance and luminance conditions were run separately, and the order of the five isoluminance and four luminance conditions was randomized.

### Model and Model Implementation

**Figure 4:**
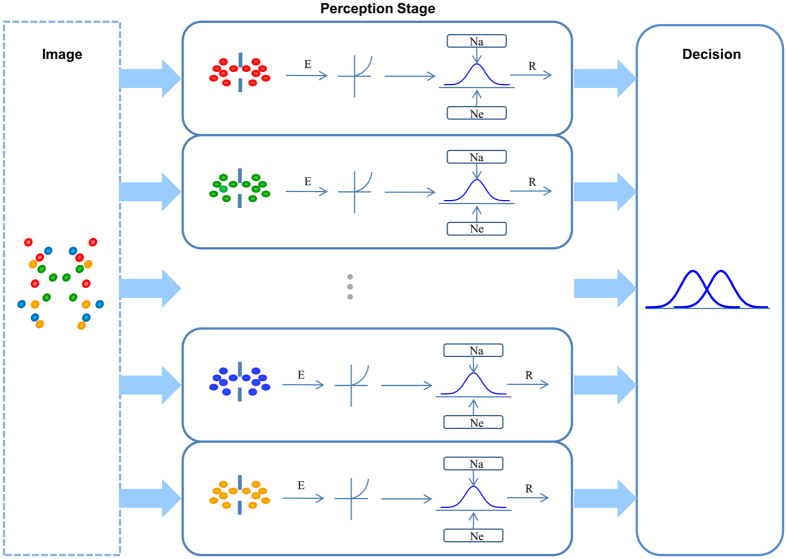
Diagram of the chromatic symmetry detection model.

**Figure 5:**
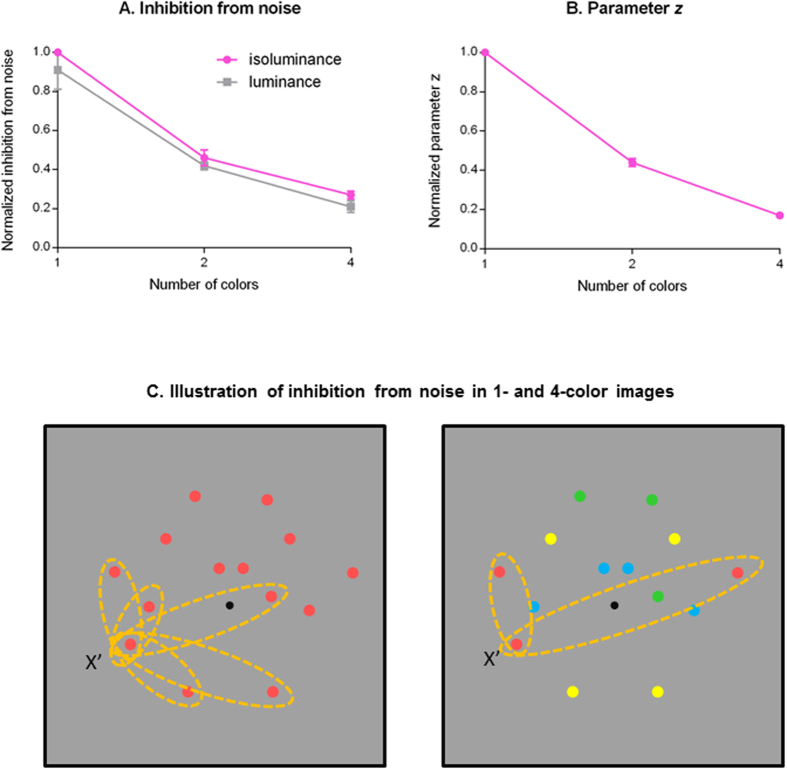
Model parameters illustration. (**A**) The amount of the normalized inhibition from noise in different conditions. The pink symbols represent the normalized inhibition for the isoluminance conditions averaged across three observers, and the gray symbols represent that for luminance conditions. (**B**) The amount of the normalized constant *z* in different conditions. The pink symbols represent the normalized *z* value for the 1-, 2-, and 4-color conditions, averaged across two types of color modulations and three observers. (**C**) The illustration of inhibition from noise in 1- and 4-color images. The number of the possible candidates each noise dot can pair with decreases when the number of the colors increases. The yellow dashed ovals represent the possible pairs a red noise dot x’ can form in the image. The red noise dot x’ can pair with all the dots in the 1-color image while it can only pair with two dots in the 4-color image.

**Figure 6:**
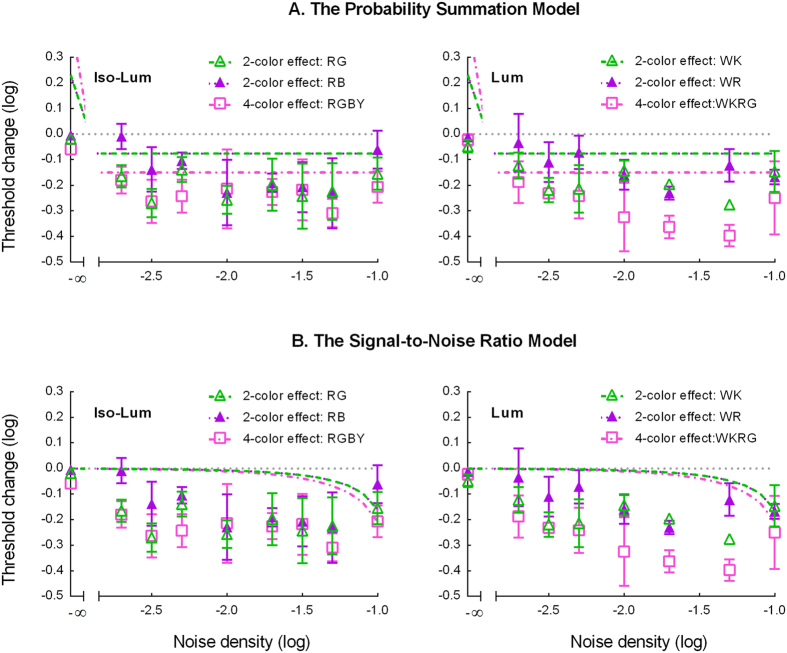
The predictions of different models. (**A**) The probability summation model. (**B**) The signal-to-noise ratio model. See text for the details.

This model is an extension of the symmetry detection model proposed by Chen and Tyler^6^, which incorporates earlier symmetry detection models^1^,^6^,^8^,^9^,^10^,^11^,^12^,^13^,^14^,^15^,^18^,^19^,^20^,^21^ with normalization processes and nonlinear summation to account for symmetry detection with multiple channels. This model contains two stages: a perception stage and a decision stage (Fig. 4). The perception stage concerns multiple color-selective symmetry channels sensitive to colored symmetric patterns, whose responses are limited by both internal and external noise. The second stage, the decision stage, concerns a higher-order mechanism which monitors these channels to determine whether the image is symmetric.

### Perception stage

In the first step of the perception stage, there is a band of color-selective symmetry channels. Each color-selective symmetry channel contains a register that analyzes spatial variations of a certain color in an image and is excited whenever there is a corresponding spatial variation at two locations in the visual field that are equidistant from a symmetry axis (a symmetry pair). The excitation from all symmetry pairs about a given symmetry axis is linearly summed to produce the excitation of that color-selective symmetry register. That is, the excitation of a symmetry channel is proportional to the number of symmetric pairs of a particular color in an image.

For a color-selective symmetry channel, each image can be divided into four components: the symmetry component of its preferred color, the symmetry component of non-preferred colors, the noise (non-symmetry) component of its preferred color, and the noise component of the non-preferred colors. Only the symmetry component of the preferred color produces excitation. In our experiment, the symmetry component was provided by the target. While there were possible coincidental symmetry pairs in the noise mask, their number was much smaller than that of the target and thus they had a negligible effect. Note that colors used in this study were deliberately selected such that the presence of one color would not affect symmetry detection in another color^22^. That is, each color is processed by a symmetry channel that is not sensitive to another color used in the experiment. Thus, for each symmetry register, we only need to consider the color selected by that register. Hence, as derived by Chen and Tyler^6^, the excitation of a register is proportional to the density of dots of a particular color in the target. Formally, the excitation of the *j*-th symmetry channel to the symmetric target or signal, *E*_*j*_, is


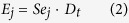


where *Se*_*j*_ is the excitatory sensitivity of the *j*-th symmetry channel to the target, while *D*_*t*_ is the density of the symmetric target (or signal) to that channel.

All of the image components produce excitation in all relevant channels, which in turn send inhibition to each symmetry channel. The inhibition received by the *j*-th symmetry channel from the channel excited by *i*-th image component is


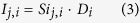


where *Si*_*j,i*_ is a positive value serving as the inhibitory sensitivity of the *j*-th channel to the image component *i*, while *D*_*i*_ is the density of the *i*-th image component.

The response of the *j*-th symmetry channels is the excitation of the corresponding symmetry register, *E*_*j*_, raised by a power *p*, and then divided by a divisive inhibition term *I*_*j*_ plus an additive constant *z*. That is,


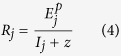


where *I*_*j*_ is the summation of a non-linear combination of the inhibition from the channel itself, and other channels excited by different image components, to the *j*-th channel. This divisive inhibition term *I*_*j*_ can be represented as


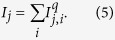


This divisive inhibition model or normalization model is widely accepted in contrast discrimination^46^,^47^,^48^,^49^,^50^,^51^,^52^,^53^ and here we apply this model to symmetry detection.

The second stage, the decision stage, concerns the decision criterion for integrating the responses of the multiple channels. The output of the perception stage is sent to the decision stage, which monitors all the relevant channels whose color selectivity matches that of the image. The observer detects a symmetric pattern if the maximum response of all monitored channels to an image is greater than the response to a noise pattern by an amount that exceeds the level of noise in the system^26^.

When there are *n* channels involved in the task, the maximum response of these channels can be described by a distribution whose mean approximates a fourth-power summation over these *n* channels^25^,^27^,^28^,^54^, that is,


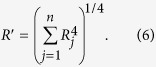


The contribution of each channel to visual performance is limited by both the internal noise in that channel and the external noise provided by the noise patterns. The variability of the internal noise, *σ*_*a*_^*2*^, is a constant for all symmetry channels. The variability of external noise, *σ*_*e*_^*2*^, is proportional to the square of the density of the noise mask, that is, *σ*_*e*_^*2*^ = *v* . *D*_*m*_^*2*^ in which *v* is a scalar constant and the index *m* denotes the noise mask. Pooled together, in each channel the standard deviation of the response distribution is


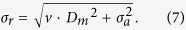


In a 2IFC task, there are two intervals, one of which contains a symmetric target and a noise mask (target interval) while the other contains a balancing control and a noise mask (non-target interval). The decision variable, *d*′, is the difference between the response to the image with the symmetric target and mask (*R*_*t+m*_) and the response to the balancing control and mask (*R*_*nt+m*_) divided by the standard deviation of the max distribution, which can be approximated by the standard deviation in each channel times a constant γ, where γ = 1, 0.82, and 0.70 for the 1-, 2- and 4-color conditions respectively^54^. That is,


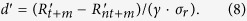


The threshold occurs when *d*′ reaches unity.

Note that, in Chen and Tyler^6^, the task of the observers was to detect symmetry with no prior knowledge about which one of four possible symmetry axis orientations would be presented in each trial. Hence, while an observer had to monitor four different symmetry channels (one for each symmetry axis orientation), only one channel would respond to the stimulus in each trial. In our experiment, however, multiple channels could respond to the stimulus simultaneously in each trial.

We set the excitatory sensitivity of each color-selective channel to be the same for all conditions since the contrast of each pattern was always set at three times the contrast detection threshold. We also set the inhibitory sensitivity of *j*-th channel to the image component of the non-preferred colors to be zero since it has been shown that the presence of a noise pattern in one color has little, if any, influence on the detection of the symmetric pattern in another color^22^. In addition, we used a typical value of 2 for the power for the divisive inhibition input *q*, as in the original normalization symmetry detection model proposed by Chen and Tyler^6^. Hence, there are four free parameters, two *Si (Si*_*t*_, the inhibitory sensitivity of *j*-th channel to symmetric target of its preferred color, and *Si*_*m*_, to noise of its preferred color), *z* and *p*, for each condition. However, we empirically found that setting *Si*_*t*_ and *p* to be the same for all conditions, while varying *z* with the number of colors and *Si*_*m*_ with the type of color modulation (e.g., isoluminance and luminance) and the number of colors in a multicolor pattern, provided a good fit to the data. Thus, instead of 36 free parameters (4 parameters x 9 conditions), our model contained only 11, given those constraints. Further details of the model implementation are described in Supplementary Method.

## Additional Information

**How to cite this article**: Wu, C.-C. and Chen, C.-C. The Integration of Color-Selective Mechanisms in Symmetry Detection. *Sci. Rep.* **7**, 42972; doi: 10.1038/srep42972 (2017).

**Publisher's note:** Springer Nature remains neutral with regard to jurisdictional claims in published maps and institutional affiliations.

## Supplementary Material

Supplementary Information

## Figures and Tables

**Table 1:** Fitted model parameters.

			CCW	CPY	HYC
*Se*_t_*			1000	1000	1000
*Si*_*t*_			13.41	20.92	27.93
*Si*_*m*_					
	1-color	(R,B)	1504	1684	885
	2-iso-color	(RG,RB)	1491	1298	875
	4-iso-color	(RGBY)	1834	1571	936
	1-lum-color	(W)	1655	1443	694
	2-lum-color	(WK,WR)	1336	1307	776
	4-lum-color	(WKRG)	1596	1230	658
*z*	1-color		1.65	2.17	1.51
	2-color		0.68	1.04	0.63
	4-color		0.27	0.33	0.29
*p*			1.84	1.76	1.61
q*			2	2	2

^*^Fixed value, not a free parameter.

**Table 2:** The coordinates of the color space and chromoluminance cone contrast space of the color.

Name	Coordinates in DKL space	Coordinates in the cone contrast space (C_L_, C_M_, C_S_)
White (W)	(0°, 90°)	[0.577, 0.577, 0.577]
Black (K)	(0°, −90°)	[−0.577, −0.577, −0.577]
Red (R)	(0°, 0°)	[0.416, −0.909, 0.000]
Blue (B)	(90°, 0°)	[0.000, 0.000, 1.000]
Green (G)	(180°, 0°)	[−0.416, 0.909, 0.000]
Yellow (Y)	(270°, 0°)	[0.000, 0.000, −1.000]
