# Supplementary material for: The Integration of Color-Selective Mechanisms in Symmetry Detection
Source: Sci Rep. 2017 Feb 23;7:42972. doi: 10.1038/srep42972 (PMC5322352; doi:10.1038/srep42972)
Supplement: Supplementary Information [file srep42972-s1.pdf]

Supplementary Information

The Integration of Color-Selective Mechanisms in Symmetry Detection

Chia-Ching Wu, Chien-Chung Chen

Supplementary Discussion

At high noise densities, the noise experienced by the visual system and, in turn, the symmetry detection threshold is dominated by the external noise provided by the noise mask[1]. The threshold is achieved when the signal to noise ratio (i.e., the ratio of symmetry pairs to unpaired dots) reaches a certain level. Suppose that an observer can detect symmetry with a certain probability if there are *k* symmetry pairs in a 1-color pattern with *k'* unmatched dots. Given the fourth-power law[1,2-4], we would expect the observer to detect symmetry in a two-color pattern with a signal-to-noise ratio of 0.84**k/2* in each color. Sum up the color components, and the threshold for the 2-color pattern at a given noise density should be about 0.84 times, or 0.076 log units, lower than for the 1-color pattern. Similarly, the threshold for the 4-color pattern should be about 0.15 log units lower than for the 1-color pattern.

Supplementary Method

Threshold Measurement

To ensure that our result would not be contaminated by a difference in salience or sensitivity to early visual features, we set the contrast of each color component at three times its detection threshold. We used a temporal 2IFC paradigm to measure the contrast threshold for symmetry detection in the colors used in the experiment for each observer. In each trial, a vertical symmetric target was randomly presented in one of the two intervals while a balancing control was presented in the other. The density of both images was 1%. The duration was 233ms and the inter-stimulus interval (ISI) was 600ms. An audio tone indicated the beginning of each interval. The observers' task was to judge which interval contained a vertical symmetric pattern. An audio feedback was provided for the response. The PSI threshold-seeking algorithm[5] was used to determine the contrast level for each trial and to measure the threshold at 75% correct level. There were 40 trials for each threshold measurement. The threshold was an average of 4 to 10 measurements. The order of the tested colors was randomized.

With this measurement, we got the contrast threshold for each color for each observer. In the experiment, we set the contrast of each color at three times its threshold for each observer based on this measurement.

Model Implementation

Our experiment used a 2IFC design, in which one interval contained a symmetric target and a noise mask (target interval), while the other interval contained a balancing control and a noise mask (non-target interval). In an *n*-color condition, the target, balancing control, and noise mask contained an equal density of dots in each

color. Let the density of the symmetric target and balancing control in the image be D_t', and that of the noise mask be D_m'. In the n-color condition, the density of the symmetric target or balancing control of a specific color is thus expressed as D_t'/n and that of the noise mask as D_m'/n.

The j-th color-selective symmetry channel is excited by a symmetric target or signal of its preferred color. The noise of its preferred color inevitably contains spurious symmetric pairs which occur completely by chance. These spurious pairs should also produce its excitation. However, the density of these pairs is the square of the density of the noise, which is negligible compared to either the symmetric target or noise itself. Therefore, for the sake of simplicity, we did not take the effect of these pairs into consideration in our model. Hence, in the target interval, including the target itself and the mask, the excitation of the j-th symmetry channel in equation (2) was expressed as

$$E'_{j,t+m} = Se_j \cdot \frac{D_t'}{n}, \tag{S1}$$

while in the non-target interval, including the balancing control and the mask, $E'_{j,nt+m}$, was zero.

All the image components produce inhibition to the j-th channel, including the symmetric target or signal of its preferred color, the symmetric component of its non-preferred color, the noise component of its preferred color, and the noise component

of its non-preferred color. Here, we set the inhibitory sensitivity to a channel from the image components of its non-preferred color as zero, since our previous paper showed that the presence of dots of one color had little, if any, influence on detecting a symmetric pattern of another color[6]. We also used a typical value of 2 for the power q for the divisive inhibition term[7-9]. Hence, equation (5) in the target interval was expressed by

$$I_{j,t+m} = \left(Si_t \cdot \frac{D'_t}{n} \right)^2 + \left(Si_m \cdot \frac{D'_m}{n} \right)^2, \tag{S2}$$

while in the non-target interval it was expressed by

$$I_{j,nt+m} = \left(Si_m \cdot \frac{D'_t + D'_m}{n} \right)^2, \tag{S3}$$

where the subscript of inhibitory sensitivity Si denotes the contribution from each stimulus component; t denotes the target or symmetry component; and m, the noise component.

We fixed the internal noise in the j-th channel, σ_a^2 in equation (7), at 1, because there was no difference in the TvD functions among the three 1-color conditions in our data. The external noise to the channel, σ_e^2 in equation (7), was set to be $\nu \cdot (D'_m$

$/n)^2$, since the density of the external noise is D'_m/n. Hence, the standard deviation of the response distribution in each channel in equation (7) was expressed by

$$\sigma_r = \sqrt{v \cdot \left(\frac{D'_m}{n}\right)^2 + 1}, \tag{S4}$$

In addition, since we used a typical value of 2 for the power for the divisive inhibition input q, we can combine inhibition and noise terms and simplify the model by approximating the response of the individual channel in equation (4) in the target interval by

$$R'_{j,t+m} = \frac{\left(Se_j \cdot \frac{D_t'}{n}\right)^p}{\left(Si_t \cdot \frac{D_t'}{n}\right)^2 + \left(Si_m \cdot \frac{D_m'}{n}\right)^2 + z'}, \tag{S5}$$

and the response in the non-target interval, $R'_{j,nt+m}$, by zero, where the parameters Si_t, Si_m, z' and p are free parameters in the model. The decision variable, d', therefore becomes

$$d' = \frac{R'_{t+m}}{\gamma}, \tag{S6}$$

where γ= 1, 0.82, and 0.70 for the 1-, 2- and 4-color conditions respectively[10].

In practice, we also fixed Se_j at 1000 for all conditions as an anchor point, since we set the contrast of each color at three times its symmetry detection threshold. Hence, there are 4 free parameters in each condition.

Reference

1. Pelli, D. C. Uncertainty explains many aspects of visual contrast detection and discrimination. *J Opt Soc Am A*. **2**, 1508–1532 (1985).
2. Graham, N., Robson, J. G. & Nachmias, J. Grating summation in fovea and periphery. *Vision Res*. **18**, 815–825 (1978).
3. Quick, R. F. A vector magnitude model of contrast detection. *Kybernetik*. **16**, 65–67 (1974).
4. Tyler, C. W. & Chen, C. C. Signal detection theory in the 2AFC paradigm: Attention, channel uncertainty and probability summation. *Vision Res*. **40**, 3121–3144 (2000).
5. Kontsevich, L. L, & Tyler, C. W. Bayesian adaptive estimation of psychometric slope and threshold. *Vision Res*. **39**, 2729–2737 (1999).
6. Wu, C. C. & Chen, C. C. The symmetry detection mechanisms are color selective. *Sci Rep*. **4**, 10.1038/srep03893 (2014).
7. Foley, J. M. Human luminance pattern-vision mechanisms: Masking experiments require a new model. *J Opt Soc Am A Opt Image Sci Vis*. **11**, 1710–1719 (1994).

8. Foley, J. M, & Chen, C. C. Pattern detection in the presence of maskers that differ in spatial phase and temporal offset: threshold measurements and a model. *Vision Res*. **39**, 3855–3872 (1999).

9. Hegger, D. J. Half-squaring in responses of cat striate cells. *Vis Neurosci*. **9**, 427–443 (1992).

10. Chen, C. C. & Tyler, C. W. Accurate approximation to the extreme order statistics of Gaussian samples. *Commun Stat Simul Comput*. **28**, 177–188 (1999).
